# Association Between Arterial Stiffness and Measures of Autonomic Dysfunction in People With Chronic Kidney Disease

**DOI:** 10.1177/20543581231213798

**Published:** 2023-11-21

**Authors:** Rachelle Davies, Natasha Wiebe, Andrew Brotto, Michael K. Stickland, Branko Braam, Stephanie Thompson

**Affiliations:** 1 Division of Nephrology, Department of Medicine, University of Alberta, Edmonton, Canada; 2 Division of Pulmonary Medicine, Department of Medicine, University of Alberta, Edmonton, Canada

**Keywords:** chronic kidney disease, autonomic dysfunction, arterial stiffness, blood pressure variability, systolic dipping

## Abstract

**Background:**

Autonomic nervous system (ANS) dysfunction and vascular stiffness increase cardiovascular risk in people with chronic kidney disease (CKD). Chronic elevations in sympathetic activity can lead to increased arterial stiffness; however, the relationship between these variables is unknown in CKD.

**Objective:**

To explore the association between measures of autonomic function and arterial stiffness in patients with moderate-to-severe CKD.

**Methods:**

This study was a prespecified secondary analysis of a randomized controlled trial. This included the following measures: 24-hour ambulatory blood pressure (BP), carotid-femoral and carotid-radial pulse wave velocity (PWV), and postexercise heart rate recovery (HRR). We used mixed effect linear regression models with Bayesian information criteria (BIC) to assess the contribution of ANS measurements.

**Results:**

Forty-four patients were included in the analysis. Mean carotid-femoral and carotid-radial PWV were 7.12 m/s (95% CI 6.13, 8.12) and 8.51 m/s (7.90, 9.11), respectively. Mean systolic dipping, calculated as percentage change in mean systolic readings from day to night, was 10.0% (95% CI 7.79, 12.18). Systolic dipping was independently associated with carotid-radial PWV, MD −0.09 m/s (95% CI −0.15, −0.02) and had the lowest BIC.

**Conclusions:**

Systolic dipping was associated with carotid-radial PWV in people with moderate-to-severe CKD; however, there was no association with carotid-femoral PWV. Systolic dipping may be a feasible surrogate of ANS function, as the association with carotid-radial PWV was consistent with the minimal clinically important difference (MCID). Future studies are needed to define the relationship between ANS function, arterial stiffness, and CV events over time in people with CKD.

## Abrégé

**Contexte:**

Le dysfonctionnement du système nerveux autonome (SNA) et la rigidité artérielle augmentent le risque d’événements cardiovasculaires chez les personnes atteintes d’insuffisance rénale chronique (IRC). Les élévations chroniques de l’activité sympathique peuvent accroître la rigidité artérielle, mais on ne connaît pas le lien entre ces variables en contexte d’IRC.

**Objectif:**

Explorer l’association entre les mesures de la fonction autonome et la rigidité artérielle chez les patients atteints d’IRC modérée ou grave.

**Méthodologie:**

Cette étude était l’analyze secondaire prédéfinie d’un essai contrôlé randomisé. Mesures incluses: le suivi de la pression artérielle (PA) ambulatoire sur 24 heures, la vitesse de l’onde de pouls (VOP) carotido-fémorale et carotido-radiale, et la récupération de la fréquence cardiaque (HRR—Heart Rate Recovery) après l’effort. Nous avons eu recours à des modèles de régression linéaire à effets mixtes avec critères d’information bayésiens (BIC — Bayesian Information Criteria) pour évaluer la contribution des mesures du SNA.

**Résultats:**

Quarante-quatre patients sont inclus dans l’analyze. La valeur moyenne des VOP carotido-fémorale et carotido-radiale était respectivement de 7,12 m/s (IC 95 %: 6,13 à 8,12) et de 8,51 m/s (IC 95 %: 7,90 à 9,11). La chute systolique moyenne, calculée en pourcentage de variation entre les valeurs systoliques moyennes du jour et de la nuit, était de 10,0 % (IC 95 %: 7,79 à 12,18). La chute systolique a été indépendamment associée à la VOP carotido-radiale, avec un écart moyen de -0,09 m/s (IC 95 %: -0,15 à -0,02) et a présenté les plus faibles BIC.

**Conclusion:**

La chute systolique a été associée à la VOP carotido-radiale chez les personnes atteintes d’IRC modérée ou grave, mais aucune association n’a été observée avec la VOP carotido-fémorale. La chute systolique pourrait être un substitut de la fonction du SNA, car l’association avec la VOP carotido-radiale était cohérente avec la différence minimale cliniquement importante (DMCI). D’autres études sont nécessaires pour mieux définir la relation entre la fonction du SNA, la rigidité artérielle et les événements cardiovasculaires au fil du temps chez les personnes atteintes d’IRC.

## Introduction

The risk of cardiovascular disease (CVD) in people with chronic kidney disease (CKD) is significantly higher than in the general population.^1^ Arterial stiffness is increased in people with CKD and is independently associated with cardiovascular (CV) risk.^2-4^ Autonomic nervous system (ANS) dysfunction is prevalent in CKD and contributes to the progression of CKD and increased CVD risk.^5,6^ The hallmark of ANS dysfunction is increased sympathetic nervous system (SNS) activity and blunted parasympathetic nervous system function.^7^ Elevations in SNS activity acutely increase blood pressure (BP), but also play a role in the long-term regulation of BP^8^ by altering vascular function (eg, reduced nitric oxide bioavailability^9^ and increased reactive oxygen species). An association between markers of SNS activity and vascular stiffness has been reported in other in chronic disease populations;^10-13^ however, there is limited data in people with non-dialysis-dependent CKD. In one study, coexisting comorbidities explained the relationship between stiffness and SNS measures.^14^

Sympathetic nervous system activity can be assessed directly with muscle sympathetic nerve activity (MSNA) and plasma catecholamines.^15^ However, there are several other noninvasive, clinically available measures that are established surrogates of ANS function, including measures of blood pressure (BP) variability^16^ and heart rate recovery (HRR) from exercise. BP variability is defined as the short-term fluctuations in BP measured during 24-hour ambulatory blood pressure monitoring (ABPM).^17^ Accordingly, the daytime standard deviation and coefficient of variation (CV) of systolic BP are potential screening tools for assessing ANS function.^18,19^ One-minute HRR, defined as the change in heart rate from peak exercise to 1-minute postexercise, is also a powerful prognostic indicator of CV risk and events^20,21^ and can be obtained following cardiopulmonary exercise testing (CPET).^22^ Chronotropic incompetence^23^ is another potential surrogate of autonomic dysfunction obtained from CPET that independently predicts all-cause mortality,^24^ and is defined as the attenuated heart rate response to exercise (achieving <80%-85% of the maximum predicted heart rate [MPHR])^25^. However, the association between the above measures and arterial stiffness is unknown in CKD.

The primary objective of this study is to determine the association between non-invasive measures of ANS, (Table 1), and arterial stiffness, as measured by pulse wave velocity (PWV). Our second objective was to determine which of these candidate measures is most strongly associated with stiffness. We hypothesized that higher BP variability, attenuated HRR and MPHR, and lower cardiorespiratory fitness are associated with greater arterial stiffness in patients with moderate-to-severe CKD.

**Table 1.** Candidate Measures of Autonomic Function.

Component of ANS	Measures	Definition
Blood pressure variability	Mean 24-hour SBP and DBP (mm Hg)	Mean systolic and diastolic blood pressure readings taken over 24 hours
	Daytime SBP and DBP (mm Hg)	Blood pressure reading during daytime hours (6 a.m. to 10 p.m.)
	Systolic dipping (%)	The difference between daytime mean systolic pressure and nighttime mean systolic pressure expressed as a percentage of the day value. Dipping less than 10% is considered blunted.
	Standard deviation (SD) and coefficient of variation (CV) of daytime BP measures	Measures of dispersion around mean BP between 6 a.m. to 10 p.m.
Heart rate response to exercise	Heart rate recovery (bpm)	Difference in heart rate measured in beats per minute at peak to 1-minute postexercise. Less than 12 beats/min from peak after 1 minute is considered a prognostic indicator of cardiovascular risk and events.
	Maximal predicted heart rate (%)	Percentage of an individual’s age-predicted maximal heart rate achieved during the CPET
Cardiorespiratory fitness	Predicted VO_2_peak (%)	Percentage of an individual’s predicted VO_2_peak (based on height, body mass, sex and age) achieved during the CPET
	VO_2_peak (mL/kg/min)	Peak rate of oxygen consumption, defined as achieving one or more of the following criteria: 1. Respiratory exchange ratio ≥1. 2. An intensity rating of breathlessness or leg discomfort of ≥5 on the 0 to 10 modified Borg scale. 3. A peak HR no less than 10 beats/minute of the age-predicted maximum of 220-age.

ANS = autonomic nervous system; SBP = systolic blood pressure; DBP = diastolic blood pressure; BP = blood pressure; CPET = cardiopulmonary exercise test; VO_2_peak = peak rate of oxygen consumption; HR =heart rate.

## Materials and Methods

This was a prespecified secondary analysis of data from a randomized controlled trial “PAIRED” (Physical Activity in Renal Disease).^26^ Participants were eligible for inclusion if they were 18 years or older, hypertensive (defined as resting systolic blood pressure [SBP] greater than 130 mm Hg on screening measurement)^27^ and had an estimated glomerular filtration rate (eGFR) of 15 to 44 mL/min per 1.73 m^2^ on at least 2 occasions in the past year.^28^ Participants were recruited from both academic and community-based CKD clinics within the Alberta Kidney Care North program in Edmonton, Alberta. Written informed consent was obtained. The University of Alberta Research Ethics Board approved this study (Pro00078564). The study was registered with the Clinical Trials.gov registry (NCT03551119). The full details of the trial protocol have been reported elsewhere.^28^

### Assessment of Blood Pressure Variability

24-hour ABPM was completed using a validated device (OnTrak Ambulatory Blood Pressure Monitor 90,227–1; Spacelabs Healthcare, Mississauga, ON, Canada) worn on the nondominant arm for a 24-hour period. Readings were obtained at 20-minute intervals from 6 a.m. to 10 p.m. and at 30-min intervals from 10 p.m. to 6 a.m. Participants were advised to perform their regular daily routine, but not to exercise during the 24-hour ABPM period.

Mean 24-hour SBP and diastolic blood pressure (DBP); daytime SBP and DBP; and systolic dipping were reported. Systolic dipping was calculated as the difference between daytime mean systolic pressure and nighttime mean systolic pressure, expressed as a percentage of the day value. In addition, we calculated the standard deviation (SD) for 24-hour and daytime BP measures.^18^ As the SD is also correlated with mean BP, we reported the CV.

### Arterial Stiffness Measurement

Central and peripheral arterial stiffness were estimated with tonometry (Complior, Alam Medical, Saint Quentin Fallavier, France) with sensors placed at the carotid-femoral and carotid-radial arterial pulse sites. The distance between measuring sites was divided by the time difference between the upslope of the pulse-waves at each measuring site, and arterial stiffness was expressed as PWV.^29^ Mean PWV was calculated as the average of at least 10 consecutive beats to cover a full respiratory cycle.

### Cardiopulmonary Exercise Testing and Heart Rate Recovery

Participants completed a standardized maximal CPET on an upright cycle ergometer (Ergoselect II 1200 Ergoline). After 5 minutes of rest, participants began cycling at 20 Watts with an increasing stepped workload of 20 Watts every 2 minutes until volitional exhaustion. 12-lead electrocardiogram recordings (CardioSoft, GE Medical Systems) were taken at rest, during exercise, and 5 minutes into recovery. VO_2_peak was determined by breath-by-breath indirect calorimetry using a cardiorespiratory metabolic measurement system (Encore229 Vmax, SensorMedics) and recorded as the peak 20-second average VO_2_ during the final minute of exercise. Peak exercise was defined as achieving one or more of the following criteria: respiratory exchange ratio ≥1, an intensity rating of breathlessness or leg discomfort of ≥5 on the 0 to 10 modified Borg scale, or a peak HR no less than 10 beats/minute of the age-predicted maximum of 220-age.^30^ HRR was defined as the difference in HR measured in beats per minute at peak to one-minute postexercise during unloaded cycling at a slow cadence (50–60 revolutions per minute).

#### Statistical analysis

All analyses were completed in Stata/MP 17.0 (https://www.stata.com) and reported baseline descriptive statistics as counts and percentages, or medians and inter-quartile ranges, as appropriate. Mean values and 95% confidence intervals were calculated for arterial stiffness and autonomic function by averaging over all 3 time points (baseline, week 8 and 24) using a clustered sandwich estimator to account for repeated measures from the participants. Carotid-femoral and carotid-radial PWV were regressed onto potential measures of ANS function using mixed effect linear regression models; participant was modeled as a random effect. Unadjusted and adjusted associations were expressed as mean differences (ie, changes in m/s per 1 unit increases in ANS function) with 95% confidence intervals. Two-sided *P*-values < .05 were considered statistically significant. Because the sample size was small, we used a forward stepwise approach. The candidate variables for the adjusted models were intervention, timepoint, age, sex, non-Caucasian ethnicity, current smoking status, diabetes, baseline body mass index (BMI), eGFR, the number of antihypertensive medications, resting HR, and 24-hour MAP. The selection of covariates and the relative contribution of the ANS function measures were quantitatively assessed using Bayesian information criterion (BIC) estimates. As described by Raftery,^31^ the BIC is an approach to model selection that helps overcome the difficulties with *P*-values and standard model selection procedures. With several candidate independent variables (as in our current analysis with various measures of ANS function), results from standard models can be misleading. However, the BIC adds a penalty based on the number of parameters being estimated in the model, with the preferred model being that which has the minimum BIC value. Thus, we sought which unadjusted and adjusted associations had the lowest BIC.

## Results

Baseline demographics and clinical characteristics are shown in Table 2. Participants had moderate-to-severe CKD and were predominantly male, Caucasian, and had a high BMI. Additional patient characteristics are reported elsewhere.^26^

**Table 2.** Demographics and Clinical Characteristics at Baseline.

Characteristic	
N	44
Age (years)	69 [56-73]
Female	16 (36.4)
Non-Caucasian	7 (15.9)
BMI (kg/m^2^)	32 [26-35]
eGFR (mL/min*1.73 m^2^)	28 [21-37]
Smoking	3 (6.8)
Diabetes	24 (54.5)
Number of BP medications	2 [1-3]
ACEi/ARB	33 (75.0)
Beta-adrenergic blocking agents	8 (18.2)
Dihydropyridine	16 (36.4)
Diuretic	25 (56.8)
Non-Dihydropyridine	7 (15.9)
Other class	2 (4.5)
No BP medications	5 (11.4)

ACEi = Angiotensin-converting enzyme inhibitors; ARB = Angiotensin II receptor antagonists; BP = blood pressure; BMI = body mass index; eGFR estimated glomerular filtration rate.

N (%) or median (interquartile range) are reported.

Table 3 shows PWV, HRR, and measures of BP variability over the trial duration. Mean carotid-femoral and carotid-radial PWV were 7.12 m/s (95% CI 6.13, 8.12) and 8.51 m/s (7.90, 9.11), respectively. Mean 24-hour systolic BP was 128 mm Hg (95% CI 125, 131). HRR was 7 bpm (95% CI 5–9). Mean systolic dipping was 10.0% (95% CI 7.8–12.2). Table 4 shows CPET parameters at peak exercise. VO_2_peak was 17.9 mL/kg/min (95% CI 16.2–19.6), which was 77.3% (95% CI 70.5, 84.1) of their predicted VO_2_peak. Participants achieved 88.9% (95% CI 85.3, 92.5) of their MPHR. The percentage of tests that were completed during which the participant both (1) exercised to volitional exhaustion and (2) met VO_2_ peak criteria was 90% (95% CI 81–95), value not indicated in table. Five tests were terminated by physician due participant meeting ECG criteria (asymptomatic). Five tests were terminated by the exercise physiologist/research staff due to participant inability to maintain cycling cadence above 50 revolutions per minute, with a mean respiratory exchange ratio of 1.2 (95% CI 1.18, 1.23) and a mean Borg rating of perceived exertion of 8.0 (95% CI 7.5, 8.5).

**Table 3.** Measures of Arterial Stiffness and Candidate Measures of Autonomic Function Averaged Across Follow-Up.

Measure	Mean [95% CI]
*Arterial stiffness*	
Carotid-femoral PWV (m/s)	7.12 [6.13-8.12]
Carotid-radial PWV (m/s)	8.51 [7.90-9.11]
*Candidate measures of autonomic function*	
HRR (bpm)	7 [5-9]
24-hour SBP (mm Hg)	128 [125-131]
24-hour DBP (mm Hg)	70 [68-73]
24-hour SBP SD (mm Hg)	15 [14-16]
24-hour SBP CV (%)	12 [11-13]
24-hour DBP SD (mm Hg)	10 [9-10]
24-hour DBP CV (%)	13 [12-15]
Daytime SBP (mm Hg)	132 [129-136]
Daytime DBP (mm Hg)	73 [70-76]
Daytime SBP SD (mm Hg)	13 [12-14]
Daytime SBP CV (%)	10 [9-11]
Daytime DBP SD (mm Hg)	8 [8-9]
Daytime DBP CV (%)	11 [10-12]
Systolic dipping (%)	10.0 [7.8-12.2]

CI = confidence interval; CV = coefficient of variation; DBP = diastolic blood pressure; HRR = heart rate recovery; PWV = pulse wave velocity; SBP = systolic blood pressure; SD = standard deviation.

**Table 4.** CPET Parameters at Peak.

Parameter	Mean [95% CI]
VO_2_ (L/min)	1.619 [1.420-1.818]
Predicted VO_2_ (%)	77.3 [70.5-84.1]
VO_2_ (mL/kg/min)	17.91 [16.28-19.55]
RQ	1.205 [1.180-1.230]
Work (Watts)	107.13 [93.50-120.75]
METS	5.13 [4.66-5.59]
VE (L/min)	65.2 [57.2-73.2]
VT (L)	2.054 [1.783-2.325]
RR (breaths/min)	32.9 [30.2-35.6]
PETO_2_ (mm Hg)	107 [105-108]
PETCO_2_ (mm Hg)	33.3 [31.7-35.0]
SpO_2_ (%)	94.7 [92.9-96.5]
HR (bpm)	128.7 [122.7-134.7]
MPHR (%)	88.9 [85.3-92.5]
SBP (mm Hg)	179 [174-185]
DBP (mm Hg)	75 [71-78]
Borg RPE	8.0 [7.5-8.5]

DBP = diastolic blood pressure; HR = heart rate; METS = metabolic equivalent of task scores; MPHR = maximal predicted heart rate; PETCO_2_ = end-tidal partial pressure of carbon dioxide; PETO_2_ = end-tidal partial pressure of oxygen; RPE = rating of perceived exertion; RQ = respiratory quotient; RR = respiratory rate; SBP = systolic blood pressure; SpO_2_ = oxygen saturation; VE = minute ventilation; VO_2_ = rate of oxygen consumption; VT = tidal volume.

**Figure 1.**
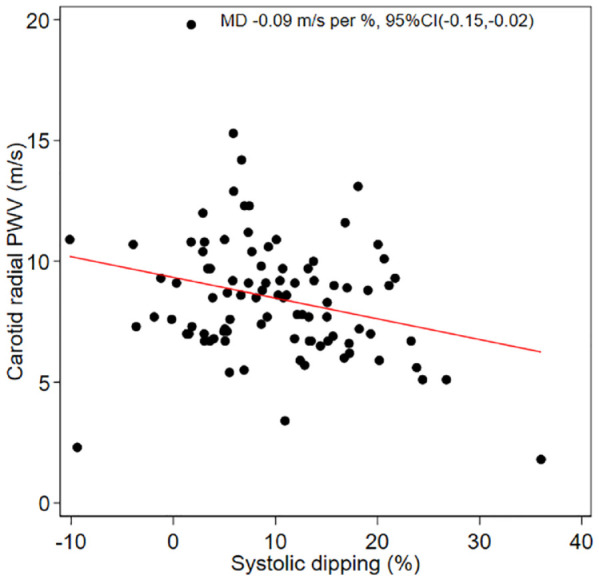
Adjusted association between systolic dipping and carotid-radial pulse wave velocity (PWV).

Tables 5 and 6 show unadjusted and adjusted associations between ANS function measures with carotid-femoral and carotid-radial PWV, presented as mean differences (changes in m/s per 1-unit increase). Systolic dipping had the strongest association with carotid-radial PWV in both the unadjusted model −0.08 m/s (95% CI −0.15, −0.02) (BIC = 453) and in the adjusted model −0.09 m/s (95% CI −0.15, −0.02) (BIC = 450), that is, for every 1% increase in systolic dipping, carotid-radial PWV decreased by an average of 0.09 m/s (shown in Figure 1). Systolic dipping was not significantly associated with carotid-femoral PWV.

**Table 5.** Unadjusted Associations of Autonomic Function Measures with Arterial Stiffness.

Autonomic Function Measure	Carotid-femoral PWV (m/s) MD	95% CI		BIC	Carotid-radial PWV (m/s) MD	95% CI		BIC
24-hour SBP (mm Hg)	0.03	[−0.04,	0.09]	528	0.02	[−0.02,	0.06]	467
24-hour SBP SD (mm Hg)	0.12	[−0.10,	0.34]	527	−0.11	[−0.26,	0.03]	465
24-hour SBP CV	0.09	[−0.20,	0.37]	528	−0.18	[−0.36,	0.00]	464
Systolic dipping (%)	−0.02	[−0.13,	0.08]	**517**	−0.08	**[−0.15,**	**−0.02]**	**453**
Daytime SBP (mm Hg)	0.02	[−0.04,	0.09]	549	0.01	[−0.03,	0.05]	479
Daytime SBP SD (mm Hg)	0.23	[−0.01,	0.46]	525	0.01	[−0.15,	0.16]	468
Daytime SBP CV	0.27	[−0.07,	0.60]	526	0.00	[−0.22,	0.22]	468
24-hour DBP (mm Hg)	−0.01	[−0.11,	0.10]	528	−0.01	[−0.07,	0.05]	468
24-hour DBP SD (mm Hg)	0.18	[−0.10,	0.46]	527	−0.12	[−0.31,	0.06]	466
24-hour DBP CV	0.14	[−0.07,	0.36]	527	−0.09	[−0.23,	0.05]	466
Daytime DBP (mm Hg)	−0.01	[−0.10,	0.09]	550	−0.02	[−0.08,	0.04]	479
Daytime DBP SD (mm Hg)	0.26	[−0.05,	0.57]	526	−0.03	[−0.23,	0.17]	468
Daytime DBP CV	0.19	[−0.06,	0.44]	521	−0.01	[−0.18,	0.15]	464
24-hour MAP (mm Hg)	0.02	[−0.08,	0.13]	528	0.02	[−0.05,	0.08]	468
Predicted VO_2_peak (%)	−0.01	[−0.06,	0.03]	564	0.00	[−0.02,	0.03]	484
VO_2_peak (mL/kg/min)	0.01	[−0.17,	0.19]	569	0.04	[−0.06,	0.15]	487
MPHR (%)	−0.01	[−0.07,	0.06]	569	−0.03	[−0.02,	0.06]	485
HRR (bpm)	0.01	[−0.47,	0.50]	569	−0.23	[−0.52,	0.07]	486
Diabetes	**2.03**	**[0.28,**	**3.78]**	564	0.34	[−0.71,	1.39]	488
BMI (kg/m^2^)	−0.12	[−0.27,	0.04]	567	0.01	[−0.08,	0.10]	488

BMI = body mass index; CI = confidence interval; CV = coefficient of variation; DBP = diastolic blood pressure; HRR = heart rate recovery; MAP = mean arterial pressure; MD = difference of means; MPHR = percent of maximal predicted heart rate; PWV = pulse wave velocity; SBP = systolic blood pressure; SD = standard deviation; VO_2_peak = peak rate of oxygen consumption. Significant associations and the lowest BICs are bolded.

**Table 6.** Adjusted Associations of Autonomic Function Measures with Arterial Stiffness.

Autonomic Function Measure	Carotid-femoral PWV (m/s) MD	95% CI		BIC	Carotid-radial PWV (m/s) MD	95% CI		BIC
24-hour SBP (mm Hg)	0.04	[−0.03,	0.10]	524	0.01	[−0.03,	0.05]	466
24-hour SBP SD (mm Hg)	0.15	[−0.06,	0.36]	523	−0.08	[−0.22,	0.06]	465
24-hour SBP CV	0.11	[−0.16,	0.38]	525	−0.13	[−0.30,	0.05]	464
Systolic dipping (%)	−0.03	[−0.13,	0.07]	**514**	−**0.09**	[−**0.15,**	−**0.02]**	**450**
Daytime SBP (mm Hg)	0.01	[−0.09,	0.11]	528	0.00	[−0.06,	0.07]	471
Daytime SBP SD (mm Hg)	**0.27**	**[0.05,**	**0.49]**	520	0.05	[−0.10,	0.19]	466
Daytime SBP CV	**0.32**	**[0.01,**	**0.64]**	522	0.07	[−0.14,	0.29]	466
24-hour DBP (mm Hg)	0.06	[−0.04,	0.17]	524	−0.01	[−0.07,	0.04]	466
24-hour DBP SD (mm Hg)	**0.31**	**[0.04,**	**0.58]**	521	−0.11	[−0.28,	0.07]	465
24-hour DBP CV	0.19	[−0.02,	0.39]	522	−0.08	[−0.21,	0.06]	465
Daytime DBP (mm Hg)	0.05	[−0.08,	0.19]	528	−0.05	[−0.13,	0.03]	470
Daytime DBP SD (mm Hg)	**0.37**	**[0.08,**	**0.66]**	519	−0.02	[−0.20,	0.17]	466
Daytime DBP CV	0.21	[−0.02,	0.44]	516	−0.02	[−0.17,	0.14]	460
24-hour MAP (mm Hg)	0.07	[−0.03,	0.17]	524	0.01	[−0.06,	0.07]	466
Predicted VO_2_peak (%)	0.00	[−0.04,	0.04]	524	0.01	[−0.02,	0.04]	466
VO_2_peak (mL/kg/min)	0.05	[−0.11,	0.22]	528	0.05	[−0.05,	0.16]	470
MPHR (%)	0.02	[−0.04,	0.08]	528	0.02	[−0.01,	0.06]	467
HRR (bpm)	0.10	[−0.34,	0.54]	528	−0.25	[−0.55,	0.05]	468
Diabetes	1.07	[−0.66,	2.79]	527	0.36	[−0.66,	1.38]	470
BMI (kg/m^2^)	−0.03	[−0.18,	0.12]	528	−0.01	[−0.11,	0.08]	470

BMI = body mass index; CI = confidence interval; CV = coefficient of variation; DBP = diastolic blood pressure; HRR = heart rate recovery; MAP = mean arterial pressure; MD = difference of means; MPHR = percent of maximal predicted heart rate; PWV = pulse wave velocity; SBP = systolic blood pressure; SD = standard deviation; VO_2_peak = peak rate of oxygen consumption. Associations adjusted for age, sex, ethnicity, BMI, eGFR, smoking, diabetes, resting heart rate, and 24-hour MAP. Significant associations and the lowest BICs are bolded.

Diabetes was associated with carotid-femoral PWV in the unadjusted model, 2.03 m/s (95% CI 0.28, 3.78); however, the association was not significant after adjustment. 24-hour DBP SD, daytime SBP SD and CV, and daytime DBP SD were associated with carotid-femoral PWV 0.31 m/s (95% CI 0.04, 0.58), 0.27 m/s (95% CI 0.05, 0.49), 0.32 m/s (95% CI 0.01, 0.64), and 0.37 m/s (95% CI 0.08, 0.66), respectively.

## Discussion

In this study, we explored the association between clinical measures of ANS function and central and peripheral stiffness in people with CKD. Our analysis revealed that systolic dipping, a measure of the day to night change in systolic BP, was most highly associated with PWV, both in the unadjusted and adjusted models. Specifically, for every 1% increase in systolic dipping, there was a 0.09-m/s decrease in carotid-radial PWV. Furthermore, measures of daytime BP variability were associated with carotid-femoral PWV in the adjusted model, which supports our hypothesis that patients with greater BP variability (as a surrogate measure of ANS function) would have greater arterial stiffness.

Our finding that systolic dipping is associated with measures of vascular stiffness is supported elsewhere in the literature. In a previous study of men and women with normal BP, high-normal BP, and stage 1 hypertension, there was an association between non-dipping and attenuated reduction of norepinephrine (NE) and epinephrine (EPI) in non-dippers compared with dippers.^32^ In people with CKD, higher MSNA was associated with reduced systolic dipping and endothelial dysfunction.^33^ Jeong et al^34^ demonstrated that individuals with CKD who had higher MSNA burst incidence also had higher nighttime BP and non-dipping patterns (81 ± 13 vs 67 ± 13 bursts/100 HR, *P* = .019). Non-dipping was also associated with endothelial dysfunction, flow-mediated dilation (FMD) (1.7 ± 1.5 vs 4.7 ± 1.9%, *P* < .001) in non-dippers compared to dippers.^34^ One of the potential mechanisms to explain this relationship is nocturnal hypoxia induced by sleep apnea,^35^ which is prevalent among people with CKD.^36^ Intermittent hypoxia has been associated with increased inflammation, oxidative stress, and sympathetic activity, leading to increased blood pressure and heart rate as well as endothelial dysfunction.^37^ In a recent study,^38^ individuals who had more severe sleep apnea also had greater arterial stiffness, particularly in those with nocturnal non-dipping BP patterns. Obstructive sleep apnea is an important link between hypertension, SNS activity, and arterial stiffness;^39^ however, further study in people with CKD is needed.

The role of pressure independent mechanisms in mediating the relationship between SNS activity and vascular stiffness remains unclear. A traditional mechanistic explanation of the association between blood pressure and arterial stiffness is that arterial stiffness precedes increases in central pulse pressure and SBP.^29^ However, recent evidence suggests SNS activity may modulate arterial stiffness independently of changes in BP.^7,40^ A review of cross-sectional studies in healthy men and women showed increased MSNA was directly associated with increased arterial stiffness after adjustment for age and BP.^41^ In healthy individuals, Faconti et al^40^ demonstrated a dissociation between changes in BP and PWV. Lower limb venous occlusion led to a 1.8 mm Hg (95% CI 0.3, 3.4) reduction in MAP, yet aortic and carotid-femoral PWV increased acutely by 0.8 m/s (0.2, 1.4) and 0.7 m/s (0.3, 1.1). In another study of healthy adults,^42^ lower body negative pressure had no significant effect on HR, BP, stroke volume, cardiac output, or total peripheral resistance, yet MSNA burst frequency, total MSNA, and carotid-femoral PWV all increased significantly. It is important to note, however, that these studies included healthy individuals and measured short-term changes in PWV from interventions that cause reflex sympathetic activation, rather than the chronic effects of elevated sympathetic activity. Although the pressure-independent relationship remains unclear in CKD, we found a significant association between carotid-radial PWV and systolic dipping that remained after adjustment for MAP and anti-hypertensive use.

While the increase in heart rate during exercise is considered a function of parasympathetic withdrawal and SNS activation, the decrease in heart rate postexercise involves reactivation of the parasympathetic nervous system. Thus, blunted HRR is thought to reflect sympathetic over-activation. Participants in our study had a mean HRR of 7.1 bpm (95% CI 4.9–9.3), which falls below the <12 bpm cut-off that has been used to predict mortality,^20^ and is lower than that reported in another study of people with stage 3 and 4 CKD^43^ (16.3 ± 9.3 bpm). On average, our study sample achieved 87.3% (95% CI 81.5, 93.1) of the MPHR, suggesting borderline chronotropic incompetence. An important consideration, however, is the potential effect of beta blocker use on HRR, which we did not include in our adjusted model due to nonsignificance.^44^ In a study of patients with known or suspected coronary artery disease,^45^ HRR was attenuated in patients using beta blockers. Conversely, a study of patients with hypertension showed no effect of beta blocker use on HRR.^46^ Others have also shown HRR retains its prognostic value independent of beta blocker use.^20,47^ Although an attenuated HRR alone might suggest elevated SNS activity in our study sample, whether it contributes to or is a consequence of stiffness remains unknown. Yang et al^48^ showed HRR was inversely associated with ultrasound carotid stiffness index after treadmill exercise with adjustment for established cardiovascular risk factors, resting SBP, and metabolic equivalent of task scores, but not with carotid stiffness index at rest. HRR was also weakly associated with brachial-ankle PWV; however, it is important to note these findings were in young, normotensive participants and may not be applicable to people with CKD.

In contrast to other studies in CKD,^49,50^ we did not find an association between VO_2_peak and PWV. Although we acknowledge this could be due to sample size, it is important to consider the lack of association could be attributed to the uniformly low cardiorespiratory fitness (CRF) of our study sample, mean VO_2_peak of 17.9 mL/kg/min, which is lower than the baseline VO_2_peak in several other studies of patients with non-dialysis CKD.^49,51-54^ Other work has shown peak peripheral oxygen extraction is a more important determinant of VO_2_peak in CKD^55^ and that PWV does not change in CKD despite significant improvements in VO_2_peak.^56^ We also reported different associations between peripheral versus central measures of arterial stiffness and ANS function. Previous studies have focused primarily on central PWV,^2^ and whether elevated SNS activity disproportionally affects peripheral stiffness is an area for future study.

### Strengths and Limitations

We included central and peripheral measures of arterial stiffness using PWV and reported an association between systolic dipping and carotid-radial PWV, including adjustment for resting HR and MAP. Previous studies comparing ANS function and vascular stiffness have not adjusted for these variables and are an important consideration for understanding the mechanistic relationship between vascular stiffness and ANS function.^40^ The association we found between systolic dipping and carotid-radial PWV was consistent with the minimal clinically important difference (MCID) for PWV (1 m/s).^57^ For every 1% increase in systolic dipping, there was a 0.09 m/s decrease in carotid-radial PWV, ie, for a 10% increase in systolic dipping, there was a 0.9 m/s decrease in carotid-radial PWV. 24-hour ABPM is also an established, readily available clinical tool for measuring BP in CKD, thus systolic dipping would be a relatively simple measure of ANS function to obtain. However, our study also has important limitations. First, we cannot infer the associations between variables determine causality. We cannot exclude residual confounding due to bias from confounding by indication, but this was mitigated by adjusting for number of antihypertensives. Second, we used indirect measures of ANS function, and we did not specifically measure SNS activity (eg, MSNA or circulating catecholamines). However, given the complexity of the ANS, there is no singular, gold standard test that accurately represents ANS function as whole; BP variability and HRR are therefore both practical and valid surrogates of ANS function and CV risk. Third, we did not attempt to quantitate vascular calcification, which is present in moderate-to-severe CKD and may contribute to arterial stiffening and reduced baroreflex sensitivity.^13^ Medial calcification in the artery wall may alter baroreceptor function and could explain the differences we observed between central and peripheral PWV. Finally, while the patients in our study had low cardiorespiratory fitness and low kidney function, they had high-normal levels of arterial stiffness and modestly elevated SBP, thus we cannot say whether the associations we found would exist in people with higher SBP and arterial stiffness.

### Future Directions

Describing the factors related to ANS function could provide greater insight into the mechanisms that contribute to CVD in people with CKD. Longitudinal studies enrolling people at earlier stages of CKD with repeated measures of aortic stiffness and SNS activity are required to better understand these mechanistic relationships. However, large scale studies measuring MSNA are less practical; thus, to facilitate the advancement of this research, identifying valid, non-invasive surrogates of ANS function is needed. It is also undetermined whether non-pharmaceutical interventions such as exercise or lifestyle modifications can adequately attenuate SNS activity in CKD. Future studies using randomized designs should evaluate whether interventions known to reduce SNS activity, such as exercise, are effective in CKD and whether these autonomic adaptations result in improved vascular stiffness.

## Conclusions

This exploratory analysis revealed systolic dipping was associated with increased carotid-radial stiffness in people with moderate-to-severe CKD. Although it is highly desirable to identify measures of autonomic function in this patient population, there is not a universal gold-standard measure of autonomic function, and thus assessments may be limited by what is clinically available. Systolic dipping warrants further investigation as it is a potential non-invasive surrogate for SNS activity and can be obtained clinically. Systolic dipping may then be a useful target for treatment with exercise and lifestyle interventions to examine whether it is both modifiable and may improve cardiovascular risk and mortality. Future studies are also needed to describe the mechanisms that underlie the relationship between ANS function, vascular stiffness, and clinical outcomes in CKD.
